# Intestinal Strangulation

**Published:** 1890-02-15

**Authors:** 


INTESTINAL STRANGULATION.

Mr. A. J. Barker, surgeon to the University College Hospital, reports an interesting case of acute intestine strangulation due to a band; with active tubercular peritonitis; laparatomy; and cure (*Bri. Med. Jour.* Jan. 18th). A young woman was admitted with severe symptoms of intestinal obstruction. There had been no action of the bowels nor flatus for three days, and vomiting had been frequent. The abdomen was uniformly distended although not tense, but a little fuller over Poupart's ligament on the right. The hidden onset of the attack, with early vomiting and complete obstruction, suggested mechanical strangulation. But evidence of peritonitis was against this diagnosis, pointing to perforation of the vermiform appendix, as did also the extra fulness in the right iliac fossa. Rupture of any other part of the intestines appeared to be excluded by the absence of collapse and the duration of the symptoms. An exploration of the cæcum was determined upon, and if nothing were found there an exploration of the abdomen by median incision. A two-inch cut was made obliquely downwards and inwards, about an inch to the right of the anterior superior spine of the ileum. Some small intestine presented at the opening, but nothing abnormal could be felt by the finger. The small intestine was thickly studded with tubercles. A median incision below the umbilicus for four inches was then made. The small intestines were found distended and thickly covered with tubercles. On passing the finger down to the right side of the pelvis a round pocket was entered upon, produced by a twist of the mesentery. The gut at this spot was fixed to the brim of the pelvis by a strong band of lymph, which was torn through. A little higher up the intestine was marked with a transverse constriction, evidently caused by being crossed by this band. Above this groove there was distension, while below the intestine was small and empty. A little below there was also a kink in the gut due to an adhesion of the peritonæal surfaces around a tubercle. The mass was cut through and its cheesy contents squeezed out, the kink being thus relieved. All obstruction being overcome the wound was flushed with hot water, and the peritonæum sponged dry. Several drachms of iodoform emulsion were then poured into both wounds among the intestines, and the abdomen closed by silk sutures. No drain tube was inserted. For some twelve hours after the operation vomiting occurred. The treatment consisted of enemata of hot water and brandy, peptonised suppositories of beef and milk, with two minims of morphine solution *quartis horis*. Vomiting recurred several times, and on the fifth day three offensive fluid motions passed. On the eighth day there was nausea, but no vomiting, after which an uninterrupted convalescence. All symptoms of tubercular peritonitis disappeared, and the abdomen was normal. Mr. Barker remarks that the family history was good, and he opines that the acute disease started with softening and rupture of a caseous mesenteric gland into the serous cavity. "If we suppose the patient to have received tubercular matter with her food while in fair health—for instance, through infected milk—the whole process is not difficult to explain. The *materies morbi* would be taken up by the surface of the intestines and carried to the nearest lymphatic glands. . . . Once the peritoneum becomes the seat of tubercle, the tendency to form bands of adhesion is great. Whether or not the patient might have ultimately recovered from the tubercular process without operation, the latter seems to have contributed to the rapid cure." Mr. Barker thinks it probable that the iodoform and glycerine emulsion aided in arresting the tubercular process.

